# Associations between the physical activity levels of fathers and their children at 20 months, 3.5 and five years of age

**DOI:** 10.1186/s12889-017-4545-8

**Published:** 2017-07-05

**Authors:** Adam D. Walsh, David Crawford, Adrian J. Cameron, Karen J. Campbell, Kylie D. Hesketh

**Affiliations:** 1 Institute for Physical Activity and Nutrition (IPAN), School of Exercise and Nutrition Sciences, Deakin University, 1 Gheringhap Street, Geelong, 3220 VIC Australia; 2 School of Health and Social Development, Deakin University, Burwood, Australia; 3 Global Obesity Centre, Deakin University, Burwood, Australia

**Keywords:** Fathers, Physical activity, Young children

## Abstract

**Background:**

Early childhood (under five years of age) is a critical developmental period when children’s physical activity behaviours are shaped and when physical activity patterns begin to emerge. Physical activity levels track from early childhood through to adolescence with low levels of physical activity associated with poorer health. The aims of this study were to examine cross-sectional and longitudinal associations between the physical activity levels of fathers and their children at the ages of 20 months, 3.5 and 5 years, and to investigate whether these associations differed based on paternal body mass index (BMI) and education.

**Methods:**

The Melbourne Infant Feeding Activity and Nutrition Trial (InFANT) Program was a cluster randomized-controlled trial delivered to pre-existing first-time parent groups. Physical activity levels of fathers and their first-born children were assessed using the Active Australia Survey and ActiGraph accelerometers respectively. Cross-sectional associations between father and child physical activity behaviours were assessed at each time point. Longitudinal associations between father and child physical activity were also investigated from child age 20 months to both 3.5 and 5 years. Additional stratified analyses were conducted based on paternal BMI and paternal education as a proxy for socioeconomic position (SEP). Data from the control and interventions groups were pooled and all analyses adjusted for intervention status, clustering by first-time parent group and accelerometer wear time.

**Results:**

Physical activity levels of fathers and their children at child age 20 months were not associated cross-sectionally or longitudinally at child age 3.5 and 5 years. Positive associations were observed between light physical activity of healthy weight fathers and children at age 3.5 years. Inverse associations were observed for moderate/vigorous physical activity between fathers and children at age 5 years, including between overweight/obese fathers and their children at this age in stratified analyses.

**Conclusions:**

There were no clear associations between the physical activity of fathers and children. Future research should include the use of more robust measures of physical activity among fathers to allow in-depth assessment of their physical activity behaviours. Investigation of well-defined correlates of physical activity in young children is warranted to confirm these findings and further progress research in this field.

## Background

Early childhood (under 5 years of age) is a critical developmental period when children’s physical activity behaviours are shaped and when physical activity patterns begin to emerge [1]. Physical activity levels track from early childhood through to adolescence [2] with low levels of physical activity associated with poorer health including increased coronary heart disease risk [3] and increased adiposity [4, 5]. Despite the commonly held perception that young children are innately active [6], evidence, although varied, suggests that many young children fail to meet physical activity guidelines [7]. Additionally, physical activity levels have been reported to decline over time with this decline beginning prior to adolescence [8]. Collectively this demonstrates the importance of understanding not only the level of physical activity in young children, but also the potential influences on physical activity over time.

Parent and child physical activity levels have consistently been reported to be positively associated from pre-school age [9–13] through to adolescence [14–17]. However, studies that specifically consider the relationships between parents and pre-school children’s physical activity do not commonly assess fathers’ influence separately, or when fathers are included, they are in the minority (or results are pooled). It is therefore currently difficult to accurately determine associations between fathers and their young children’s physical activity. In their review of the correlates of pre-school children’s physical activity, Hinkley and colleagues noted that more active parents tended to have more active children [9]. However, only one of the 24 included articles examined the independent influence of fathers’ physical activity levels, that of Moore and colleagues who observed that 4 to 7 year old children of active fathers were 3.5 times more likely to be active than children with inactive fathers [13]. It is also worth noting that two studies in the review examined the association between paternal BMI and pre-school children’s physical activity, finding an inverse association [18, 19].

A subsequent review by Bingham and colleagues [20], included an additional five studies that investigated paternal influences on children’s physical activity separately to maternal influences. Taylor and colleagues, in their longitudinal study of 244 pre-school children and their parents, reported that parental activity (mothers and fathers) was weakly positively correlated with the activity of their three and four-year-old children. After adjustment for confounders (sex, weight status and awake time of child and mothers’ activity), only fathers’ activity remained as a predictor of child activity [21]. Work by Yamamoto and colleagues [22] (*n* = 649) and Vorwerg and colleagues [23] (*n* = 92) investigated associations between fathers’ BMI and preschool children’s physical activity with neither study reporting any associations. This is in contrast to early work by Sallis and colleagues who observed an inverse association between fathers’ BMI and child moderate physical activity [19] and Finn and colleagues, who, in their cross-sectional work investigating factors associated with U.S preschool children’s physical activity, also described an inverse relationship between paternal BMI and child daily activity counts [18].

Work by Beets and colleagues examined the effect of father-child involvement and neighbourhood characteristics with young children’s (*n* = 10,694) physical activity. They described positive associations with between children’s physical activity and father-child time and children’s physical activity and family time undertaking sport together [24]. Lastly, Burgi and colleagues, in their investigation of associations between parental education, parental work status and children’s physical activity, (*n* = 542) observed no differences in child physical activity irrespective of paternal (and maternal) education or paternal work status [25]. This is in contrast to work by Yang and colleagues, who, in their longitudinal investigation of SEP as a predictor of children’s physical activity, observed that 9 year-old boys whose fathers were more highly educated participated in more sport than children of lesser educated fathers [26].

Since the Hinkley and Bingham reviews, a small number of further studies have been published on the topic of physical activity in fathers and their young children. Cantell and colleagues, in their study of the physical activity relationships between 54 Canadian pre-school children and their parents, described significant positive associations between fathers’ and children’s weekend physical activity [27]. Vollmer and colleagues also reported positive associations between paternal and child vigorous physical activity in their cross-sectional study of 150 U.S fathers of preschool children [28]. The limited number of these studies precludes firm conclusions about the associations between the physical activity levels of fathers and their young children independent of maternal influences being drawn.

As children’s physical activity begins to decline from around school entry age, [8] exploration of individual parental (i.e. mother and father) influences on the physical activity of younger children is particularly important. The level of parental influence on child behaviours differs markedly between the preschool and school years. Exploration of the determinants of pre-school physical activity, including the relationship with SEP and parental body weight, may lead to recognition of which families are at increased risk of their children not meeting physical activity guidelines and the subsequent influence on poorer health outcomes. In turn this may contribute to a better understanding of what families need so as to encourage physical activity in their young children.

The present study aimed to examine whether fathers’ physical activity levels were associated cross-sectionally with the physical activity levels of their children at 20 months, 3.5 and 5 years of age. It also examined longitudinal associations between father’s physical activity when children were 20 months old and their children’s physical activity between child age 20 months and both 3.5 and 5 years. Finally, whether these associations differed based on fathers’ education (as a proxy for SEP) or BMI was also assessed.

## Methods

### Study design

The Melbourne Infant Feeding, Activity and Nutrition Trial (InFANT) Program (ISRCTN81847050, registered 7 January 2008) was a cluster-randomized controlled trial (RCT) of a child obesity prevention intervention undertaken within pre-existing groups of first time parents, which has been described in detail previously [29, 30]. Briefly, a two-stage random sampling design was used involving 62 groups of first-time parents that were selected from 14 local government areas (across all socio-economic areas) within a 60 km (37 mile) radius of Deakin University in Burwood, Victoria, Australia. Random allocation to either control or intervention arms occurred for consenting groups. Inclusion criteria were English literacy and a minimum of eight parents in the consenting groups (or six parents in low socio-economic position area groups).

### Study sample

Exclusion criteria for this study included single parent families (*n* = 8), non-first time parents (*n* = 14), same sex couples (*n* = 1), and families where fathers did not complete a questionnaire at baseline (*n* = 58). The baseline sample consisted of 418 father-child dyads. Depending on the time point considered, follow-up physical activity data were absent or insufficient for 135–232 fathers and 122–284 children did not meet the minimal accelerometer wear-time criteria. Consequently, the final sample comprised 282 father-child dyads at child age approximately 20 months, 133 father-child dyads at child age 3.5 years and 140 father-child dyads at child age 5 years. To minimize the effect of any missing data, analyses were performed on the available sample at each time point. Physical activity levels of fathers and children in the intervention and control arms displayed no difference at any time point, and as such, data at each time point were pooled with analyses controlled for intervention status.

The Melbourne InFANT Program received approval from the Deakin University Human Research Ethics Committee (approval number: 175–2007) and the Victorian Government Department of Human Services, Office for Children, Research Coordinating Committee (approval number: CDF/07/1138). All participating families provided informed written consent.

### Measures

Fathers’ physical activity was reported for the previous week using the Active Australia Survey [31] at child age approximately 20 months, 3.5 and 5 years. The Active Australia Survey has been used in a number of population surveys in Australia and has been found to be suitable for use in self-administered format exhibiting reliability coefficients for time in each domain of physical activity from 0.56–0.64 and correlation between self-reported physical activity and accelerometer data for duration of MVPA of 0.52 [32]. Light physical activity was reported as duration of walking either for recreation, exercise or transport. Moderate physical activity was reported as duration of activities such as gentle swimming, social tennis and golf. Vigorous physical activity was reported as duration of vigorous gardening/heavy work that caused heavy breathing as well as duration of vigorous activities such as jogging, cycling and competitive tennis. Active Australia Survey standard scoring instructions were followed; duration for moderate and vigorous activity were summed and truncated at 840 min (14 h)/week where summed values exceeded this [31].

Physical activity of participating children was measured with ActiGraph accelerometers using 15 s epochs (Model GT1M; Pensacola, FL), when children were approximately 20 months, 3.5 and 5 years of age. The accelerometers were fitted to the children at all time points by trained researchers. Parents were provided with written and verbal instructions on care and how to refit the monitors on their child. Children wore the accelerometers over their right hip during all waking hours for 7 days, except when swimming and bathing. Non-wear time was considered to have occurred when there was 20 min of consecutive zero counts [33]. Previous work by Hnatiuk and colleagues determined that 4 days of monitoring was required to reliably estimate toddlers’ light physical activity (LPA) and moderate to vigorous physical activity (MVPA) [34]. A measurement day was considered valid when children recorded counts for 444 min, which represents non-missing counts for at least 80% of a standard measurement day [33]. Consequently, four valid days of data, including one weekend day, was set as the inclusion criterion for assessment of physical activity levels of children in this study. Accelerometer data were downloaded using ActiLife Software. Cut points of ≤100, 101–2292 and >2293 counts per minute (CPM) were used to determine daily time spent in sedentary activity, LPA and MVPA respectively [35]. These cut-points were initially validated for use in 5–8 year olds, [35] and have subsequently been shown to be acceptable for use in preschool-aged children [36]. While not validated in toddlers, similar cut-points (>2340) have been shown to have a mean difference of 1.4 min MVPA when compared with observed behaviour, underestimating physical activity [37]. The same cut-points were used at each age to allow comparison across all ages. Physical activity data for fathers were non-normally distributed at all times points for LPA and normally distributed at all time points for MVPA. Physical activity data for children were normally distributed at all time points for both LPA and MVPA.

Fathers’ demographic and socio-economic variables included education level (university education vs non-university education); weight; height; age; country of birth; and main language spoken at home. Weight and height data were self-reported with body mass index (BMI) calculated as weight (kg)/height (m^2^). Paternal BMI was dichotomised into healthy weight (BMI ≤ 24.99 kg/m^2^) and overweight/obese (BMI ≥ 24. 99 kg/m^2^) [38]. Paternal education was used as a proxy for SEP and was collapsed into two groups (university education vs. non-university education) [39].

### Statistical analyses

Cross-sectional and longitudinal associations between fathers’ and children’s physical activity levels at child age 20 months, 3.5 years and 5 years were measured using linear regression analyses. Primary analyses were adjusted for accelerometer wear time, intervention status and clustering by first-time parent group by using the vce (cluster) command in Stata. Additional regression analyses were undertaken, stratified by paternal BMI and education level. Subsequent analyses adjusting for maternal physical activity were performed but as the results were similar to unadjusted analyses, these have not been presented. A comparison of demographic variables between questionnaire responders (at child age 20 months) and questionnaire non-responders (at child age 5 years) occurred using t tests and χ2 analyses. Significance level was set at 5%. Analyses were conducted using Stata software (release 14; StataCorp LP, College Station, TX, USA).

## Results

Demographic characteristics, at each time point, for participating fathers in this study are presented in Table 1. At child age 20 months, mean paternal age was 35.8 years; mean paternal BMI was 27.9 kg/m^2^; 59.1% of fathers were non-University educated; the majority of the sample were Australian born and English was the main language spoken at home. No significant differences existed between the demographic characteristics of fathers lost to follow-up at child age 5 years when compared to those fathers retained in the study. Mean paternal LPA was 24.7, 21.1 and 26.9 min per day at child age 20 months, 3.5 and 5 years respectively. Mean paternal MVPA at the same time points was 41.1, 43.6 and 48.5 min per day respectively. Mean child LPA was 248.7, 274.1 and 276.3 min per day at child age 20 months, 3.5 and 5 years respectively. Mean child MVPA at the same time points was 26.4, 42.7 and 52.2 min per day respectively.

**Table 1:** Fathers’ characteristics at child age 20 months, 3.5 & 5 years

	20 months	3.5 years	5 years
	*n* = 282	*n* = 133	*n* = 140
Age (y), mean (SD),	35.8 (4.9)	37.6 (4.8)	38.9 (4.5)
BMI (kg/m^2^), mean (SD)	27.9 (5.5)	26.9 (3.6)	27.1 (4.0)
BMI Category (%)			
Healthy weight	32.3	30.5	31.7
Overweight	43.1	52.8	50.5
Obese	24.6	16.7	17.8
Education level, %			
Non-University	59.1	56.8	55.7
University	40.9	53.2	44.3
Country of birth, %			
Australia	78.6	75.7	74.5
Other	21.4	24.3	25.5
Language spoken at home, %			
English	97.4	97.6	97.0
Other	2.6	2.4	3.0
Physical Activity, mean (SD)			
LPA (min/week)	164.4 (190.1)	133.2 (141.9)	145.6 (178.2)
MVPA (min/week)	444.8 (290.1)	444.2 (278.2)	486.2 (298.9)

*BMI* Body Mass Index

*LPA* Light Physical Activity

*MVPA* Moderate to Vigorous Physical Activity

In cross-sectional analyses, fathers’ and children’s physical activity levels (both LPA and MVPA) were not associated at child age 20 months and 3.5 years, however an inverse association was observed between fathers’ and children’s MVPA at child age 5 years (Table 2). No associations were observed between fathers’ and children’s physical activity levels longitudinally. Associations between paternal physical activity and children’s physical activity stratified by paternal BMI and education are presented in Table 3. There were no associations observed between fathers’ and children physical activity levels at any time point when stratified by paternal education. When stratified by paternal BMI, no associations were observed at child age 20 months, however a positive association was detected between the LPA levels of healthy weight fathers and their children at child age 3.5 years, and an inverse association observed between the MVPA levels of overweight fathers and their children at child age 5 years. In further analyses stratified by child sex, (including analyses for total physical activity), the only association observed was between fathers and five-year-old boys for MVPA (β (95% CI) = −0.15 (−0.27; −0.27).

**Table 2:** Cross-sectional and longitudinal associations between fathers’ and children’s physical activity at child age 20 months, 3.5 and 5 years

Fathers’ physical activity at child age:	Child LPA			Child MVPA		
	Age 20 months	Age 3.5 years	Age 5 years	Age 20 months	Age 3.5 years	Age 5 years
20 months	−0.02 (−0.16; 0.11)	0.13 (−0.000; 0.26)	−0.05 (−0.27; 0.17)	−0.02 (−0.07; 0.02)	−0.05 (−0.12; 0.02)	0.03 (−0.06; 0.14)
3.5 years	--	0.04 (−0.25; 0.35)	--	--	−0.06 (−0.16; 0.02)	--
5 years	--	--	−0.17 (−0.44; 0.09)	--	--	**−0.10 (−0.18; −0.02)**

All values are β (95% CI)

All variables adjusted for accelerometer wear time, intervention status and clustering

Longitudinal models adjusted for children’s physical activity time at child age 20 months

Boldface indicates statistical significance of *P* < 0.05

*LPA* Light Physical Activity

*MVPA* Moderate to Vigorous Physical Activity

**Table 3:** Cross sectional associations between fathers’ and children’s physical activity at child age 20 months, 3.5 and 5 years according to paternal BMI and education

	20 months	3.5 years	5 years
	Light Physical Activity		
Fathers’ BMI			
Healthy weight	−0.03 (−0.25; 0.18)	**0.55 (0.02; 1.08)**	0.04 (−0.33; 0.43)
Overweight/obese	−0.03 (−0.20; 0.13)	−0.18 (−0.56; 0.18)	−0.35 (−0.72; 0.01)
Fathers’ Education			
University	0.03 (−0.19; 0.26)	−0.02 (−0.47; 0.42)	−0.27 (−0.56; 0.01)
Non-University	−0.05 (−0.24; 0.12)	0.14 (−0.27; 0.56)	−0.13 (−0.60; 0.33)
	Moderate to Vigorous Physical Activity		
Fathers’ BMI			
Healthy weight	−0.06 (−0.12; 0.007)	−0.10 (−0.31; 0.10)	−0.06 (−0.21; 0.07)
Overweight/obese	−0.008 (−0.06; 0.04)	−0.05 (−0.16; 0.06)	**−0.12 (−0.23; −0.02)**
Fathers’ Education			
University	0.01 (−0.09; 0.13)	−0.07 (−0.23; 0.09)	-0.12 (−0.27; 0.03)
Non-University	−0.03 (−0.08; 0.01)	−0.05 (−0.16; 0.04)	−0.05 (−0.13; 0.03)

All values are β (95% CI) unless otherwise indicated

All variables adjusted for accelerometer wear time, intervention status and clustering

Boldface indicates statistical significance of *P < 0.05*

*BMI* Body Mass Index

## Discussion

This study is one of only a few to examine the relationships between fathers’ and young children’s physical activity levels. We find no consistent evidence for any association. There was no association between the LPA levels of fathers and their children at child age 20 months and 5 years, or the MVPA levels of fathers and their children at child age 20 months and 3.5 years. These findings are in contrast to the study by Vollmer and colleagues who did observe associations between the physical activity levels of fathers’ and their pre-school children [28]. Previous reviews of correlates of physical activity in children by Sallis and colleagues, Gustafson and colleagues, Hinkley and colleagues and Bingham and colleagues described largely inconclusive results with respect to relationships between parental and child physical activity [9, 14, 20, 40].

Our finding of an inverse association between fathers’ and children’s MVPA at child age 5 years was unexpected (Table 2). Additionally, when stratifying physical activity levels by paternal BMI, this inverse association exists only between overweight/obese fathers’ and children’s MVPA at child age 5 years, but not between healthy weight fathers and children’s MVPA at child age 5 years (Table 3). Mean paternal MVPA at child age 5 years was 48 min per day which is substantially higher than the Australian national average for adults between 35 and 44 years of age (11 min per day) [41]. The observed inverse association between overweight/obese fathers’ and children’s MVPA at child age 5 years could simply be a chance finding, as it appears counter-intuitive given findings of previous work [9, 13]. It could also be due to the self-report nature of the fathers’ physical activity data in this study, which is prone to over-reporting. It is plausible, however, that MVPA in overweight/obese fathers may be occurring at the cost of child physical activity. For example, fathers could be jogging with their child in a stroller, cycling with their child in a seat on the back of a bicycle or the child may be engaging in sedentary activity such as reading, drawing or electronic game use while their father engages in active sports. Additionally, it may be that overweight/obese fathers’ MVPA is too intense for young children and as such occurs at times when children are not present (for example during the evening) thus negating any possible co-participation or benefit of physical activity modelling. This notion is supported by work by Hnatiuk and colleagues who, in their cross-sectional analysis of maternal correlates of young children’s physical activity observed an inverse association (in the evening period) between maternal MVPA and children’s LPA [42].

There was no observed longitudinal relationship between the physical activity levels of fathers at child age 20 months and their children’s physical activity levels at age 3.5 and 5 years. This finding is supported by a recent study by Abbott and colleagues where fathers’ physical activity at baseline (at child age 4.6 years) was not associated with children’s physical activity at follow-up (child age 7.6 years) despite observing associations between mothers and their son’s physical activity [43].

When stratified by fathers’ education (as a proxy for SEP), we observed no associations between paternal and child physical activity at any age. The few previous studies investigating the association between parental and child physical activity levels have not considered the potential impact of parental education on the association. Thus there is no previous research with which to compare our findings. However, given associations have been observed between education and physical activity levels in adults, [44] and between parent education and children’s physical activity levels, [45] further investigation of the potential influence of education is warranted.

Similarly, whilst parental BMI has been investigated as a correlate of child BMI, [28, 46] it has rarely been considered when examining the potential relationship between parental and child physical activity levels. Our findings, when stratifying physical activity levels by paternal BMI, are difficult to explain. We observed a positive association between paternal and child LPA at child age 3.5 years in dyads where fathers were of a healthy weight, and the previously identified inverse association between paternal and child MVPA for overweight fathers at child age 5 years (Table 3). As previously noted in proposed explanations of associations, there is a range of possible reasons we may have seen these associations. However, it is important to note that the observed null findings and mixed associations indicate there is likely to be little relationship between paternal BMI and young children’s physical activity in this study.

Our investigation was innovative in its emphasis on the relationships between fathers’ and their young children’s physical activity levels. Study strengths included the sampling of fathers from all SEP categories, incorporation of both cross-sectional and longitudinal data, and the use of objectively measured physical activity data for children. Study limitations to note include the impact on the strength of the current findings by the number of participants lost to follow-up. Sex-specific analysis may have also been limited by sample size. Self-reported physical activity data for fathers may be influenced by social desirability bias and individual interpretation for each activity intensity (contributing to measurement inaccuracy). Accordingly, the use of objective measures for fathers’ physical activity would allow more reliable estimates. The Active Australia Survey has been shown to provide acceptable levels of test-retest reliability across the population [47] and suitable for use in self-administered format [32] so the direction of the observed associations are not likely due to measurement error. Additionally, subjective measures do (potentially) provide the opportunity to explore the context/types/domains of physical activity, and thus increase understanding of physical activity behaviour. The data were drawn from an intervention cohort thus participants had different exposures based on their intervention status. However, any potential impact of this limitation is likely to be small given there was no intervention effect on the outcomes of interest. Any potential impact was further minimised by adjusting statistical analyses for intervention status. It is also acknowledged that there is much variation in recommended cut points for assessing children’s physical activity levels from accelerometer data. This is made more complex with the age range in this study. All cut-points have been shown to underestimate MVPA in the toddler age group [37]. However, the physical activity cut points chosen were the best approximation across all three age groups and allowed comparison across all ages.

## Conclusion

This study adds to the very limited literature assessing physical activity associations in father-child dyads involving children under 5 years of age. We found no consistent associations between the physical activity of fathers and their children over the first 5 years of life. The observed mixed associations with respect to direction, activity intensity and child age indicate that the physical activity relationships between fathers and their young children are complex. Future research should include the use of more robust measures of the physical activity levels of fathers to allow in depth assessment of fathers’ physical activity behaviours. Additionally, future research could also investigate parent-child proximity when objectively measuring physical activity in an effort to ascertain physical activity co-participation and/or parental modelling. Investigation of well-defined correlates of physical activity in young children are warranted to confirm these findings and further progress research in this field.

## Abbreviations

BMI: Body Mass Index
InFANT: Infant Feeding Activity and Nutrition Trial
LPA: Light physical activity
MVPA: Moderate/vigorous physical activity
RCT: Randomized Controlled Trial
SEP: Socioeconomic Position
